# 

**Published:** 1800-12

**Authors:** 


NEW MEDICAL PUBLICATIONS IN NOVEMBER.

A Compendium of the Anatomy of the Human Body, illuftrated by nearly 700 Figures, copied from the moft celebrated Authors, and from Nature, by Andrew Fyfe, 3 vol. 4to. 5l. 5s. Longman and Rees.

Differtations on Inflammation. 1. On the Laws of Animal Economy; 2. On theHiftory, Caufes, Confequences, and Cure of Simple Inflammation; 3. On the Phagedenic, and fome other Species of Inflammation; 4. On the Sporigoid Inflammation; 5. On Scrophulous Inflammation; 6. On the Cancerous Inflammation. By John Burns, Surgeon, Glafgow, 2 vol. 8vo. 14s. boards. Longman and Rees.

An Effay on Phlegmatia Dolens, including an Account of the Symptoms, Caufes, and Cure of Peritonitis Puerperalis, and Conjunctiva, &c. &c. by John Hull, M. D. 8vo. 6s. 6d. boards. Bickerftaff.

---

### To CORRESPONDENTS.

*The Editors consider themselves under much obligation to* I. G. *for his very sensible remarks. He may have observed that the last Index was more full than the two former ones; an arrangement has however been made, by means of which the future Indices will in all respects be full and satisfactory. The number of pages in the books reviewed shall be added. The succession of papers on the same subject will always be attended to, provided all which appear, are in the possession of the Editors when the Number of the Journal is put to press. The other observations of* I. G. *will not be neglected; and the Editors, in conclussion, wish him to make his promised communications, the value of which they anticipate by that of his present Letter.*

*Communications are received from Dr.* Pearson *and Mr.* Ring, *as are several Hints and Improvements, all of which we are obliged to postpone at present.*

### INDEX.

Page.

ABERDOUR on ftrictures of the urethra 387
Abforbent fyftem, Dr. Thilow on air received into it 86
Abforption, Mafcagni's, Prof. obfervations and experiments on 468
Abftinence from food and drink, Dr. Schmidtmann's remarkable account of in a girl 88
Accidents by lightning, Cit. Claubry on the ufe of alkalis in 370
Acetous ether, Cit. Sedillot on 84
Acid carbonic, contained in atmofpheric air, Humbold on 85
—— citric, in fea fcurvy, Trotter, Dr. on 154
———— Watherston on 155
—— nitric, Dr. Mitchell on 24
———— its ufe in fyphilus, new work of, Dr. Beddoes on, announced 91
———— Prof. Wurzer on the ufe of it in a cafe of lues venerea 183
———— Count Muffin Pufhkin on the decompofition of red lead in 374
———— Lichtenberg's experiments with 376
———— Gilby, Dr. on, in diabetes 205
Acid of tartar, Buckholz's method of obtaining it 181
Acid phofphoric, Suerfen's method of obtaining it 447
—— fulphuric, Kinglake, Dr. on the medicinal effects of 482
Ackermann, Prof. his differtation on wind in the inteftinal tube 556
Aconiti extractum, Dr. Gebel on, in rheumatic cardialgy 184
Æther and tinct. opii, in cramp of the ftomach, Samuel Thomas on 444
Affections convulfive, Dr. Hargens on the ufe of alkali in 183
Affection fpafmodic, Shaul on 48
Affufion cold, Schaw on 25
———— Reeve on 397
———— and the ufe of yeaft in typhus 401
Ague, Dr. Knebel's method of curing it 83
Aikin's, Meffrs. their lectures announced 283
Air, Bouffey's Dr. Refearches into its influence in caufing difeafes 449
—— received into the abforbent fyftem, Dr. Thilow on 86
——, atmofpheric, Humbold on carbonic acid, contained in 81

Page.

Alderfon, Dr. his account of the efficacy of tractors 100
Alkali, Dr. Hargens on the ufe of, in convulfive affections 183
Alkalies, Cit. Claubry on the ufe of, in accidents by lightning 370
Almanack or pocket book for Chemifts and Apothecaries 82
Amaurofis, obftinate, on the ufe of mercurius corrofivus fublimatus in 184
———— Dr. Beer on the application of cold water in *(with plate)* 179
America, fketch of the Univerfity of Cambridge, in 569
Amputation, Chauffier on 406
———— Knipe on 415
Anatomy, Theatre of, in Windmill Street, lectures announced at 282
Aneurifms, Ayrer, Dr. on 367
Angina Pectoris, Hill on 30
———— Cappe, Dr. on 222
Animal, Profeffor Blumenbach on one newly difcovered in New South Wales 83
Anthracometer, Humboldt's defcription of, *(with engraving)* 179
Apothecaries and Chemifts, almanack or pocket book for 82
Archives, northern, of natural and medical fcience, Pfaff and Dr. Scheele's 75
———— for the medical knowledge of countries 365
Arnemann, Prof. on acouftic tubes *(with an engraving)* 378
Afthma, Sugrue, Dr. on 329
Ayrer, Dr. on Aneurifms 367
Azote, Dr. Girtanner's analyfis of 185
Ballard on a peculiar fpecies of ulcers 408
Barlow on the delivery of the placenta 320
Barry, Dr. on the hot bath 146
———— on cow-pock 425
Bartley, on quackery 231
———— on delivering the placenta 233
———— on artificial mufk 235
Bath hot, Dr. Barry on it 146
—— waters, Gibbes, Dr. on 457
Batty, Dr. his lectures announced 283
Beddoe's, Dr. his new work on the ufe of nitric acid in fyphilus, announced 91
Beer, Dr. on the application of cold water in amaurofis *(with plate)* 179
Beet root, method of obtaining fugar and brandy from 2[illegible]
—— Lampfadius, Prof. his experiments on the fugar of it 366
Bell, on Scrophula 59

Bellamy on catarrhal fever 290
Belladonna, Mr. Wainwright on 5
Bell, Mr. John, his work on military furgery announced 472
Beryll Saxon, Tromfdorf, Prof. on a new earth in 84
Bills of mortality, account of deaths in for the laft three months 474
Bladder difeafed, Ford's cafe of a deceafed bladder *(with engraving)* 390
Blair, his letter to Dr. Bradley, refpecting Dr. Nifbet's cafe of cancer 569
Blane Dr. on hæmorrhage from the liver 67
Bleaching, Cit Chaptal on 469
Blood, Dr. Myer's experiment to try whether iron particles enter into it with the chyle 186
Blyth on the practice of Hippocrates 155
Blumenbach, Prof. on an animal lately difcovered in New South Wales 83
Books, Critical Retrospect of, Dr. Blane on Hæmorrhages from the Liver, 67. Mr. Rumfey on Croup, 69. Dr. Ruffell on the Co-exiftence of Small-Pox and Meafles, *ib.* Mr. Jenner's Addrefs on the Advantages of Vaccine Inoculation, 71. Prof. Dumas's Methodical Syftem of Nomenclature, &c. 73. Prof. Pfaff and Dr. Scheel's Northern Archives of Natural and Medical Science, 75. Prof. Hildebrandt's Encyclopedia of Chemiftry, 76. Doctor Van Heckeren's Differtation on morbific Offification, *ib.* Doctor Hecker's Theory of difficult Dentition in Children, 77. Docter Brandis's Theory of ditto, 79. Almanac or Pocket-Book, for Chemifts and Apothecaries, 82. Dr. Clarke on the Prefentation of the Face to the Os Pubis, 103. Mr. Home on a Cafe of Pregnancy, 165. Dr. Harnefs on the Application of Gaftrc Juice to Sores, 167. Dr. Hull's Obfervations on Mr. Simmons's Detection, &c. 168. Mr. Dunning on Vaccination, 171. Dr. Wichmann's Theory of difficult Dentition in Children, 171, 269. Mr. Lauth's Anatomy of the Mufcles, 177. Prof. Bourguet's Manual Lexicon of Chemiftry, 178. Dr. Woodville's Obfervations on the Cow-pox, 256. Mr. Fermor's Reflections on the Cow-pox. Dr. Johnftone, on Madnefs, 260. The Edinburgh Practice of Phyfic and Surgery, 262. Nofology; tranflated from the Latin of Dr. Cullen, *ib.* Dr. Bree's Practical Enquiry into difordered Refpiration, *ib.* Mr Davy's Chemical and Philofophical Researches, 265, 360, 451. Dr Prieftley, on Phlogifton, 269. Mr. Chamberlaine's Treatife on Stizolobium, *ib.* Prof. Hufeland's Remarks on the Brunonian Practce, 271. Dr. Werner's Apology for the Brunonian Syftem, 275. Mr. Scarpa on the Structure of the Bones, 276. Dr. Saunders on Mineral Waters, 350. Dr. Fowle's Treatife on Fevers in the Weft Indies, 354. Mr. Moore's Treatife on Ophthalmy, 358. Mr. Sapon on the Plague, 364. Archives for the Medical Knowledge of Countries, 365. Prof. Lampfadius's Experiments on the Sugar of the Beet, 366. Dr. Cappel on the Brunonian Syftem, 367. Dr. Ayrer on Aneurifms, *ib.* Dr. Kitaibel, &c. on the Plants of Hungary, 368 Dr. Bouffey's Refearches on the Influence of Air in caufing Difeafes, 449. Dr. Vaume on Vaccine Inoculation, 450. Travels in the Empire of Flora, *ib.* Mr. Coquebert's Defcription of new Infects, *ib.* Mr. Kentifh on Burns, 454. Doctor Gibbes on the Bath Waters, 457. Mr. Parkinfon's Hofpital Pupil, 459. A Comparative Statement of Facts and Obfervations relative to the Cow-pox, 463. S. T. Soemmering Tabula Bafeos Encephali, 552. S. T. Soemmering Icones Embryonum Humanorum, 554. Prof. Ackermann's Differtation on Wind in the Inteftinal Tube, 556 Prof. Reich's Theory and Practice of Fever, *ib.* Dr. Waterhoufe on the Kine Pox, 563. A confcious View of Circumftances, &c. refpecting Vaccine Inoculation, 567.
Bones, Scarpa on their ftructure
Bofquillon Dr. on Hydrophobia 56
Bouffey Dr. his refearches on the influence of air in caufing difeafes 449
Boulogne, account of the vaccine inoculation at 470
Bourguet's Prof. Manual Lexicon of chemiftry 178
Bradley Dr. letter to him by Dr. Denman on cow-pox 1
——— his lectures announced 282
——— Nifbet Dr. his note to 476
——— Lettfom's Dr. letter to, on the cow-pox 567
——— Blair's letter to him refpecting Dr. Nifbett's cafe of cancer 569
Brain, Gall's work on the exercife of it 90
——— and Nerves, Purton on the phyfiology of 334
Breafts, Nifbet Dr. and Mr. Oliphant's cafe of cancer of both 296
Bree, Dr. his practical enquiry into difordered refpiration 262
Bronchocele, Ring on 412
Brugnatelli, his new arrangement of mineral waters 377
Brunonian fyftem, Cappel Dr. on it 367
——— Hufeland's, Prof. remarks on it 271

Brunonian fyftem, Werner's, Dr. apology for it 275
Buniva Cit. on the epizootic difeafe of cats 373
Burns, Kentifh Dr. on 454
Calomel, Carfon Dr. on the ufe of 410
Cambridge, in New England, Waterhoufe Dr. his account of the vaccine inoculation there 471
——— in America, fketch of the Univerfity of 569
Cancer of both breafts, Dr. Nifbett's and Mr Oliphant's cafe of 296
——— Blair's letter to Dr. Bradley refpecting Dr. Nifbett's cafe of 956
Cappe, Dr. on angina pectoris 222
——— on the cow-pock 429
Cappel Dr. on the mineral waters of Dryburg, in Weftphalia 87
——— on the Brunonian fyftem 367
Cardialgy rheumatic, Dr. Gebel on the extractum aconiti in 184
Carpue, his lectures announced 283
Carfon Dr. on the ufe of calomel 410
——— his work announced on the treatment of pregnant women 471
Cats, Cit. Buniva on the epizootic difeafe of 373
Cayley Dr. on Quackery 404
Chalcedony blue, from Siberia, Prof. Tromfdorf on it 374
Chamberlain, his treatife on ftizolobium 269
——— extracts of two letters addreffed to him 567
Chantran Cit. on the mildew 372
Chaptal Cit. on bleaching 469
Charcoal, Lowitz on the purifying property of 375
Chavaffe, his cafe of diabetes mellitus 211
Chauffier on amputation 406
——— Cit. Van Mons's preparation of the new falt difcovered by him 469
Children, difficult dentition in, Dr. Hecker's theory of 77
——— Dr. Brandis's theory of 79
——— Dr. Wichmann's theory of 171, 269
Chifholm Dr. his effay on the malignant and peftilential fever of Grenada announced 284
Chyle, Dr. Myers's experiment to difcover whether iron particles enter into the blood with it 186
Chemiftry, Hildebrandt's Encyclopedia of 76
——— Profeffor Bourguet's manual Lexicon of 178
Chirurgical and medical education, Parkinfon's effay on, announced 380
Chemical and philofophical refearches, Davy's 360
Chemifts and apothecaries, almanack or pocket book for 82
Chevalier, his lectures announced 283
Clarke Dr. on the prefentation of the face to the os pubis 163
——— and Ofborn Drs. their lectures announced 282
Claubry Cit. on the ufe of alkalis in accidents by lightning 370
Clubfoot, Sheldrake on 493
Clutterbuck on tetanus 287
Coal, Prof. Parrot's experiments on 183
Coccinella feptempuncta, remarks on as a remedy for the tooth-ach 84
Combuftion human, Pieree-Aimee Lair on 45
Commiffioners of fick and wounded feamen, Vietch's letter to on vaccine inoculation 306
Confumption, Mofsman Dr. on 308
Contagion variolous, Franks on 518
Cooke, on vaccine inoculation 21
Cooper on perforation of the membrana tympani 570
Coquebert, his defcription of a new fpecies of infects 450
Correfpondents, to, 92, 188, 284, 380, 476, 572
Cowley on diflocation of the jaw 503
Cow-pox, Dr. Denman on 1
——— Lord Derby's letter to Dr. Denman on 2
——— inoculation, declaration of phyficians and furgeons refpecting it 187
——— Woodville's Dr. obfervations on 256
——— Fermor's reflections on 259
——— Cappe Dr. on 429
——— Barry Dr. on 425
——— Lettfom's Dr. letter to Dr. Bradley on 567
——— comparative ftatement of facts and obfervations relative to 463
——— Marfhall, Dr. on 386, 544
Cramp of the ftomach, Thomas on æther and tinct. opii in 444
Cranium, fracture of, Dr. Evans's cafe 346
Creffes, garden, Prince Gallitzen's experiments on the germ. of their feeds 86
Crichton Dr. his lectures announced 282
Croup, Leefon on 34
——— Rumfey on 69
——— Cuftance on 20
——— on digitalis in 420
Crowther Dr. on the induration of the veficulæ feminales 507
——— his cafe of fcorbutus 510
——— his cafe of the fatal effects of opium obviated 512
Cryftallization of platina, Count Muffkin Pufhkin on 373
Cryftals, rock of Switzerland, Girtanner's experiments on them 469
Cullen Dr. his Nofology tranflated from the Latin 262

Cure and prevention of diseases, Dr. Thomson's work on announced 472

Custance on Quackery 17

——— on yeast in typhus 19

——— on croup 20

——— on digitalis in croup 420

Cynips rosæ, Hirth on, in the cure of tooth-ach 377

Darwin Dr. Charles, his experiments on pus 50, 108, 203

Davies on the doses of medicine 160

——— on Hydrothorax 384

Davy, annunciation of his work on Gases and Respiration 91

Davy, his chemical and philosophical researches 265, 360, 451

Deafness, Dr. Wolcott's treatise on the general causes of it announced 187

Deaths of medical men 91

——— in the bills of mortality for the last three months, account of 474

Decomposition of red lead in nitric acid, Count Mussin Pushkin on 374

Denman Dr. on cow-pox 1

——— Lord Derby's letter to him on cow-pox 2

Dentition, difficult, in children, Dr. Hecker's theory of 77

——— Dr. Brandis's theory of 79

——— Dr. Wichmann's theory of 171 269

Dennison and Squire, Drs. their lectures announced 91, 283

Diabetes, Dr. Gilby on nitrous acid in 205

——— melitus, Chavasse's case of 211

Digitalis, Dr. Maclean on 127, 237

——— in croup, Custance on 420

——— Drake Dr. on 521

Discovery, Hahnemann's, of a new salt 466

Discoveries Anatomical, Sprengel Prof. his history of concluded 340

Disease, epizootic of cats 373

——— vaccine, Pegge, Sir Christopher, on the origin of 381

Diseases infantile, Woodward on 42

——— Boussey's Dr. researches on the influence of air in causing 449

——— cure and prevention of, Dr. Thomson's work on, announced 472

——— in London, Willan's Dr. observations on 472

Diseases of the Finsbury dispensary, monthly report of, 14, 105, 202, 298, 393, 488

Diseases in the Westminster hospital, account of 14, 107, 201

——— from Aug. 20, to Sept. 24 475

——— from Sept. 14, to Oct. 22 475

Diseases, account of in an eastern district from the 20th of May to the 20th of June 13

——— from 20th June to 20th July 103

——— from 20th July to 20th Aug. 201

Diseases from 20th Aug. to 20th Sept. 472

——— from 20th Sept. to 20th Oct. 473

Dislocation of the jaw, Cowley on 503

Distillation of vinegar, Simons Dr. on 411

——— of the oxygenated pomatum, Cit. Van Mons on 470

Domeier Dr. on the cure of polydipsia 304

——— on the furred tongue 422

Drake, Dr. on digitalis 521

Dropsical case, Dr. Guthrie, his remedy in one 570

Dropsy, Magennis Dr. on the cure of 212

Dysphagia, Helsham's Dr. cases on *(with engravings)* 477

Dumas Professor, his methodical system of nomenclature 73

Dunning, his definition of cow-pox

Earth, Prof. Tromsdorf on a new one in the Saxon Beryll 84

Eastern district, v. *Diseases in an eastern district*

Eckeberg's account of a blackish stone found at Ytterby in Sweden 185

Edinburgh practice of physic and surgery 262

Education medical and chirurgical, Parkinson's essay on, announced 380

Effects medicinal of sulphuric acid, Dr. Kinglake on 482

——— fatal, of opium, obviated case of by Dr. Crowther 512

Electricity Galvanic, Nicholson on 115

Empire of Flora, travels in 450

Epilepsy, Magennis Dr. on 417

——— Cit. Portal on opium in 570

Ether and tinct. opii in cure of the stomach, Thomas on 444

Erysipelas, Stokes Dr. on 301

Ether acetous, Cit. Sedillot on 84

——— acetic in rheumatic complaints, Cit. Martin on the effects of 369

Evans Dr. his case of a fracture of the cranium 346

Experiments, Dr. Charles Darwin's on pus 50, 108, 203

———, Messrs. Roupen and Van Norden's 469

——— with nitric acid, Lichtenberg's 376

——— and observations on absorption 468

——— Hermbstaedt's to obtain sugar from indigenous plants 347

——— Lampsadius's Prof. on the sugar of the beet 366

——— Lichtenberg's with nitric acid 376

——— Hermbstaedt's with the leaves and roots of the tulip tree 376

——— and observations on absorption by Prof. Mascagni 467

Experiments, Girtanner's with the rock cryſtals of Switzerland 469

———— Harrington's Dr. announced 572

Extracts from two letters addreſſed to Mr. Chamberlain, ſurgeon 567

Facts, comparative ſtate of, and obſervations relative to the cow-pox 463

Fermor, his reflections on the cow-pox 259

Fever, Reich's Prof. theory and practice of 556

Fever, ſcarlet, Dr. Hahnemann on a ſpecific remedy for 87

Fever, malignant and peſtilential of Grenada, Dr. Chiſholm's eſſay upon it, announced 284

Fever, Catarrhal, Bellamy on 290

Fevers intermitting, Dr. Plies on flores arnicæ in 87

—— in the Weſt Indies, Dr. Fowle's treatiſe on 354

Finſbury diſpenſary, monthly report of diſeaſes at, 14, 105, 202, 298, 393, 488

Flora, empire of, travels in 450

Flores arnicæ in intermitting fevers, Dr. Plies on 87

Fluids perſpirable, Dr. Mitchell on 9, 109

—— purulent, Wollaſton on the danger of inoculating with 488

Ford, his caſe of a diſeaſed bladder *(with an engraving)* 390

Fordyce Dr. his lectures announced 282

Fowle Dr. his treatiſe on fevers in the Weſt Indies 354

Fracture of the cranium, Dr. Evans's caſe of 346

———— ſkull, Veitch on 513, 572

France, vaccine inoculation at Boulogne in, account of 470

Franks on variolous contagion 518

Furred tongue, Domeier on 422

Gall, his work on the exerciſe of the brain, reviewed 90

Gallitzin's Prince, on the germination of the ſeeds of garden creſſes 86

Gaſes and reſpiration, Mr. Davy's work on announced 91

Gaſtrodynia, Sugrue's Dr. caſe of 228

Gebel Dr. on the extractum aconiti in rheumatic cardialgy 184

Geoghegan on ſtrangulated hernia 317

Germany, new medical publications in 284

Gilby Dr. on nitrous acid in diabetes 205

Gilleſpie Dr. on the bites of ſerpents 293

Girtanner Dr. his analyſis of azote 183

———— on the uſe of oxygen in venereal complaints 469

———— his experiments with the rock-cryſtals of Switzerland 469

Gibbes Dr. on the Bath waters 457

Glands indurated, account of an anatomical preparation being incruſted with ſaline cryſtals 184

Gmelin Prof. his experiments with the tellurium 86

Gravel and urinary ſtones, Prof. Maſcagni's obſervations on 476

Gregory Dr. his memorial announced 471

Grenada, malignant and peſtilential fever of, Dr. Chiſholm's eſſay on, announced 286

Groſe on the uſe of yeaſt and cold affuſion in typhus 401, 539

Guthrie, his caſe of hydrocephalus 219

——— Dr. his remedy in a dropſical caſe 570

Hahnemann, Dr. on a ſpecific remedy for the ſcarlet fever 87

———— on the diſcovery of a new ſalt 466

Hamilton, Prof. on the nature of electric matter 85

Hammick, his lectures announced 571

Hare-lip, Purton on the operation for 336

Hargens, Dr. on the uſe of alkali in convulſive affections 183

Harneſs, Dr. on the application of gaſtric juice to ſores 167

Harrington, Dr. his experiments, &c. announced 572

Hecker, Dr. his theory of difficult dentition in children 77

Helſham, Dr. his caſes of dyſphagia, *(with engravings)* 477

Hermbſtaedt, Dr. his experiments and obſervations on the extraction of ſugar and ſyrup from indigenous plants 246, 347

————, Prof. his experiments with the leaves and roots of the tulip tree 376

Hernia, Ward on the treatment of 37

————, ſtrangulated, Geoghegan on 317

Hildebrandt's Encyclopedia of Chemiſtry 76

Hildenbrand, Dr. on two remedies to prevent and cure hydrophobia 89

Hill on angina pectoris 30

Hints for improvements 380

Hirſh on the cynips roſæ in the tooth ach 377

Hippocrates, Blyth on the practice of 151

Home on a caſe of pregnancy 165

Hoſpital, St. Thomas's and Guy's lectures announced at 280

————, plan of clinical lectures at 281

————, London, lectures at, announced 282

Hufeland, Prof. his remarks on the Brunonian practice 271

Huggan, Dr. on vaccine inoculation 343

Hull, Dr. his obſervations on Simmons's detection 168

————, his Eſſay on Phlegmatia Dolens announced 283

Hull, Dr. his Epitome of the Nofologia Methodica of Sauvages, announced 284
Humboldt, on carbonic acid contained in atmofpheric air 85
————, his experiments on the illumination of rotten wood *ib.*
Humboldt's defcription of his Anthracometer, *(with an engraving)* 179
Hungary, plants of, Kitaibel Dr. on 368
Hydrophobia, Bofquillon Dr. on 56
————, Hildenbrand Dr. on two remedies to prevent and cure it 89
Hydrothorax, Davies on 384
Iceland, obfidian foffil of, Prof. Tromfdorf's analyzation of it 377
Icones Embryonum Humanorum, S. T. Soemmering 554
Improvements, hints for 380
Inoculating with purulent Fluids, Wollafton Dr. on the danger of 488
Induration of the Veficulæ Seminales, &c. 507
Inoculation, Dr. Juncker on 141
————, fmall-pox in 1746, Dr. Pierce Dod on 7
Inoculation vaccine, Cooke on 21
————, Dr. Juncker on 144
————, Jenner's addrefs on the advantages of 71
————, account of at Paris 90
————, declaration of furgeons and phyficians refpecting it 187
————, on the introduction of, at Paris 253
————, official report from the houfe for, at Vaugirard in Paris 255
————, Inftitution, Warwick Street, London, notice from *ib.*
————, Veitch's letter to the commiffioners of fick and wounded feamen, on 306
————, Leefe on 337
————, Wilfon Dr. on 340
————, Huggan Dr. on 343
————, Vaume Dr. on 450
————, account of, at Boulogne in France 470
————, at Cambridge in New England, account of 471
————, a conscious view of circumftances refpecting 567
————, teftimonial in favor of 570
Infects, new, Coquebert's defcription of 450
Intelligence, Medical and Phyfical, v. Medical and Phyfical Intelligence 83
Inteftinal tube, Prof. Ackermann's differtations on wind in 556
Iron, Dr. Meyer's experiments to try whether its particles enter into the blood with the chyle 186
Ifchuria, Richmond on 331
————, Helfham's Dr. cafes of 479
Jaw, its diflocation, Cowley on 503
Jenner's addrefs on the advantages of vaccine inoculation 71
Johnftone, Dr. on madnefs 260
Jörden's, Dr. ointment as a remedy in chronic ophthalmies 96
Juice, gaftric, Dr. Harnefs on the application of, to fores 167
Juncker, Dr. on inoculation 141
————, on the extirpation of fmall-pox 143
————, on vaccine inoculation 144
Kali common, Lowitz on the nature of 375
Kelfon, Mr. on vaccine inoculation 21
Kentifh on Burns 454
Kine-pox, Dr. Waterhoufe on 563
Kinglake, Dr. in explanation to Dr. Sherwen relative to fox-glove 83
————, on delivering the placenta 156
————, on the medicinal effects of fulphuric acid 482
————, in anfwer to Mr. S. Thomas 487
Kirkland, Dr. his third volume announced of medical furgery 471
Kitaibel, Dr. on the plants of Hungary 368
Knebel, Dr. his method of curing the ague 83
Knipe on amputation 415
Knowledge medical and chirurgical, tranfactions of a fociety for the improvement of 67
———— of countries, archives for 365
Lampfadius, Prof. his experiments on the fugar of the beet 366
Lauth, his anatomy of the mufcles 177
Lead, red, on its decompofition in nitric acid, Count Muffin Pufhkin on 374
Lectures announced, Stanclift's, at the Leverian Mufeum 91
————, Dr. Dennifon's and Dr. Squires's 91, 283
————, at St. Thomas's and Guy's Hofpital 280
————, at the London Hofpital 282
————, at the Theatre of Anatomy, Windmill Street *ib.*
————, Dr. Fordyce's *ib.*
————, Dr. Pearfon's *ib.*
————, Dr. Crichton's *ib.*
————, Dr. Bradley's *ib.*
————, Drs. Ofborne and Clarke's *ib.*
Lectures, clinical, plan of at St. Thomas's and Guy's Hofpitals 280
Lectures announced, Dr. Batty's 283
————, Dr. Pole's *ib.*
————, Mr. Pearfon's *ib.*
————, Mr. Chevalier's *ib.*

Lectures announced, Mr. Carpue's *ib.*

——, Messrs. Aikin's *ib.*

——, Mr. Hammick's 571

Ledum palustre, Dr. Maton on the medicinal properties of 97

Leese, Mr. on the co-existence of small-pox and measles 29

Leeson on croup 34

Legs, old sore, on the use of semina phelandrii aquatici in the cure of 377

Lettsom, Dr. his letter to Dr. Bradley on the cow-pox 567

Leverian Museum, Stancliff's lectures at announced 91

Lichtenberg, his experiments with nitric acid 376

Lightening, Cit. Claubry on the use of alkalis in accidents by 370

Limbs, distorted, Watt's description of a new machine for curing them 93

——, Sheldrake on, in answer to Watt

Linck's, Prof. account of the pregnancy of a mule in Portugal 186

Liver, Dr. Blane on hæmorrhage from it 67

London, diseases in, Dr. Willan's observations on, announced 472

Lowitz on the nature of common kali 375

—— on the purifying property of charcoal *ib.*

Lucas on making putrid water again potable 87

Lues venerea, Prof. Wurzer on the use of nitric acid in a case of 183

Madness, Dr. Johnstone on 260

Magennis, Dr. on the cure of dropsy 212

——, on Epilepsy 417

Malformation, Simmons's case of, (*with an engraving*) 189

Mania, Dr. Crowther's case of 508

Maclean, Dr. on Digitalis 127, 237

Mammut ohioticum, Dr. Seybert relating to the bones of it 186

Marshall, Dr. on cow-pox 386, 544

Mascagni's, Prof. observations on urinary stones and gravel 467

Maton, Dr. on the medicinal properties of ledum palustre 97

Matter, electric, Prof. Hamilton on the nature of 85

Measles and small-pox, Leese on the co-existence of 29

——, Dr. Russel on the co-existence of 69

Medical surgery, Kirkland's third volume of, announced 471

Medical Publications, v. *Publications, new medical*

Medical and Physical Intelligence, 83, 183, 278, 369

Medicines, Davies on the doses of 160

—— febrifuge, Uwins on 53

Membrana tympani, Cooper on its perforation 570

Mercurius sublimatus corrosivus, on its use in obstinate Amaurosis 184

Meyer, Dr. his experiments to discover whether iron particles enter into the blood with the chyle 186

Michaelis, Dr. on a peculiar sort of tumour 445

Midwifery, Mr. Sam. Thomas on 441

Mildew, Cit. Chantran on 372

Mitchill, Dr. on the perspirable fluids 109

—— on nitric acid 24

Moore his treatise on ophthalmy 358

Monthly report of diseases at the Finsbury Dispensary, 298, v. *Diseases*

Mossman, Dr. on consumption 308

Mule, Prof. Linck's account of the pregnancy of one in Portugal 186

Muscles, Lauth's anatomy of them 177

Museum Leverian, Stancliff's lectures at, announced 91

Musk, artificial, Bartley on 235

Mussin Poushkin, Count, on the crystallization of platina 373

——, on the decomposition of red lead in nitric acid 374

Nerves and brain, Purton on the physiology of 334

Nicholson on Galvanic electricity 115

Nisbet, Dr. and Mr. Oliphant's case of cancer of both breasts 296

—— his note to Dr. Bradley 476

Noble, his treatise on ophthalmy announced 471

Nomenclature, Prof. Dumas's methodical system of 73

Nosologia Methodica of Sauvages, Dr. Hull's Epitome of it announced 284

Nosology, translated from the Latin of Dr. Cullen 262

Obsidian fossil of Iceland, Prof. Tromsdorf's analyzation of it 377

Ointment, Dr. Jörden's as a remedy in chronic ophthalmies 90

Oliphant and Dr. Nisbet's case of cancer in both breasts 296

——, his cases 545

Ophthalmies, chronic, Dr. Jörden's ointment as a remedy for 90

Ophthalmy, Moore's treatise on 358

——, Noble's treatise on announced 471

Opii, tinct. and æther, in cramp of the stomach, Thomas on 444

Opium, case of the fatal effects of, obviated, Dr. Crowther's 512

—— in epilepsy, Cit. Portal on 570

Osborne and Clarke, Drs. their lectures announced 282

Ossification, morbific, Dr. Van Heckeren's dissertation on 76

Oxygen, Girtanner on the use of in venereal complaints 469

Oxygenated pomatum, Cit. Van Mon's distillation of 479

Paris, account of the vaccine inoculation at 90
——, on the introduction of the vaccine inoculation at 253
Parkinfon, his effay on medical and chirurgical education announced 380
Parkinfon, his Hofpital Pupil 459
Parrot, Prof. his experiments on coal 185
Patella, diflocated, Waiblinger's cafe of 285
Peaal, his cafe of polydipfia 340
Pearfon, Dr. his lectures announced 228
——, Mr. his lectures announced 283
Pegge, Sir Chriftopher, on the origin of the vaccine difeafe 381
Pfaff and Dr. Scheele's northern archives of natural and medical fcience 75
Pharmacopeia, Reece's notice refpecting a new one 571
Philofophical and chemical refearches, Davy's 360
Phlogifton, Prieftley, Dr. on 269
Phofphorus, on the medicinal ufe of 164
Phyfic and Surgery, the Edinburgh Practice of 262
Phyfiology of the brain and nerves, Purton on 334
Pierce Dod, Dr. on fmall-pox inoculation in 1746 7
Pierre Aimee Lair, on human combuftion 45
Placenta, on delivering it, by Dr. Syer 3
——, by Dr. Kinglake 156
——, by Bartley 233
Placenta, Barlow on 320
Plague, Sapon on the 364
Plants, indigenous, Dr. Hermbftaedt's experiments and obfervations on the extraction of fugar and fyrup from, 246, 247
Platina, Count M. Poufhkin on a new method of forging 84
—— on the cryftallization of 373
Plies, Dr. on flores arnicæ in intermitting fevers 87
Pole, Mr. his lectures announced 283
Polydipfia, Domeier, Dr. on the cure of 304
——, Peaal's cafe of 403
Poifon, Spry's cafe of 516
Pomatum, oxygenated, Cit. Van Mons diftillation of 470
Portal, Cit. on opium in epilepfy 570
Pox, fmall, Dr. Juncker on the extirpation of 143
Pox, fmall, and meafles, Leefe on the co-exiftence of 29
—— Dr. Ruffell on the co exiftence of 69
Pox, cow, Dunning's definition of 146
—— Woodville, Dr. his obfervations on 256
Pox, cow, Feimor's reflections on 259
Practice, Brunonian, Hufeland's, Prof. remarks on it 271
—— Werner's, Dr. his apology for it 275
Pregnancy, Home on a cafe of 165
Pregnant women, Carfon's, Dr. work on the treatment of announced 471
Prieftley, Dr. on phlogifton 269
Procefs pharmaceutical 181
Publications, new, medical, 92, 188, 284, 475, 572
——, in Germany 284
Purton on the phyfiology of the brain and nerves 334
Pus, Dr. Charles Darwin's experiments on 50, 108, 203
Quackery, Cuftance on 17
—— Bartley on 231
—— Cayley on 404
Reece, his notice refpecting a new Pharmcopeia 571
Reich, Prof. his theory and practice of fever 556
Refpiration and gafes, Davy's work on announced 91
——, difordered, Bree's, Dr. practical enquiry into 262
——, Prof Abildgaard on 278
Reeve on the affufion of cold water 397
Richmond, his model of a fpeculum oculi 103
—— on Ifchuria 331
Ring on Bronchocele 412
Roupen and Van Norden's experiments 469
Ruffell, Dr. on the co-exiftence of fmall-pox and meafles 69
Salt, Hahneman's, Dr. difcovery of a new one 466
—— new, difcovered by Chauffier, Cit. Van Mon's preparation of it 469
Sapon on the plague 364
Saunders, Dr. his treatife on mineral waters 91, 350
Sauvage's Nofologia Methodica, Dr. Hull's epitome of, announced 284
Scarpa on the ftructure of the bones 276
Scheele, Dr. and Pfaff's northern archives of natural and medical fcience 75
Schmidtmann's account of a remarkable abftinence in a girl from all food and drink 88
Scorbutus, Dr. Crowther's cafe of 510
Scrofula, Bell on 59
Scurvy, fea, Dr. Trotter on the citric acid in the cure of 154
—— Watherfton on ditto 155
Sedillot, Cit. on the acetous æther 84
Semina phelandrii aquatici, on its ufe in curing old fore legs 377
Serpents, Gillefpie, Dr. on the bites of 293

Sergeant on Vaccine 505
Seybert, Dr. relating to the bones of the mammut ohioticum 186
Shaul on fpafmodic affections 48
Sheldrake on diftorted limbs, in anfwer to Watt 192
——— on the club-foot 493
———, Watt in reply to 498
Siberia, blue chalcedony from, Prof. Tromfdorf on 374
Simmons, his cafe of malformation *(with an engraving)* 189
Skull, Veitch on the fracture of 513, 572
Simons, Dr. on the diftillation of vinegar 411
Simmons's detection, &c. Dr. Hull's anfwer to 168
Soemmering, S. T. tabula bafeos encephali 552
———, icones embryonum humanorum 554
Sores, Dr Harnefs on the application of gaftric juice to 167
Speculum oculi, Reich's model of one 103
Sprengel, Prof. his hiftory of anatomical difcoveries concluded 340
Spry, his cafe of poifon 516
Squire and Dennifon, Drs. their lectures announced 91, 283
Stancliff, his lectures at the Leverian Mufeum announced 91
Stokes, Dr. on eryfipelas 301
Stomach, Thomas on æther and tinct. opii in cramp of 444
Stone, Eckeberg's account of a blackifh one found at Ytterby in Sweden 185
Stones, urinary, and gravel, Prof. Mafcagni's obfervations on 467
Strictures of the urethra, Aberdour on 387
Suerfen's method of obtaining phofphoric acid 447
Sugar and brandy, method of obtaining from the beet root 278
Sugar and fyrups, experiments and obfervati ns on extracting from indigenous plants, Dr. Hermbftaedt's 246, 347
——— of the beet, Lampfadius's Prof. his experiments on 366
Sugrue, Dr. on afthma 329
——— his cafe of gaftrodynia 228
Surgery and Phyfic, the Edinburgh Practice of 262
———, military, Mr. John Bell's work on, announced 472
Switzerland, rock cryftals of, Girtanner's experiments with 469
Syer, Dr. on delivering the placenta 3
Syphilus, Dr. Beddoes's new work on nitric acid in, announced 91
Tartar, acid of, Buckholtz's method of obtaining it 181
Tellurium, Prof. Gmelins's experiments with it 86
Tetanus, Clutterbuck on 287
Thilow, Dr. on air received into the abforbent fyftem 86
Thomas, Mr. S. on midwifery 441
——— on æther and tinct. opii in cramp of the ftomach 444
Thomfon's, Dr. work on the cure and prevention of difeafes announced 472
Tooth ach, obfervations on the coccinella feptempuncta as a remedy for 84
——— Hirfh on the cynips rofæ in the cure of 377
Tractors, Alderfon's, Dr. account of their efficacy 100
Tromsdorf, Prof on a new earth in the Saxon beryll 84
——— on the blue chalcedony from Siberia 374
——— his analyzation of the obfidian foffil of Iceland 377
Trotter, Dr. on the citric acid in fea fcurvy 154
Tubes, acouftic, Prof. Arnemann on *(with an engraving)* 378
Tulip-tree, Prof. Hermbftaedt's experiments with its leaves and roots 376
Tumour, Michaelis, Dr. on a peculiar fpecies of 445
Typhus, Cuftance on yeaft in 19
———, Grofe on the ufe of yeaft and cold affufion in 401, 539
Vaccination, Dunning on 171
Vaccine Inoculation, Cooke on 21
———, Kelfon on *ib.*
———, Jenner's addrefs on the advantages of 71
——— in Paris, an account of 90
———, on the introduction of, at Paris 253
——— houfe at Vaugirard in Paris, official report from 255
——— inftitution, Warwick Street, London, notice from *ib.*
———, Vaume, Dr. on 450
———, teftimonial in favour of 570
——— at Cambridge in New England, Dr. Waterhoufe's account of 471
——— at Boulogne, in France, account of 470
Vaccine difeafe, Sir Chriftopher Pegge on the origin of 381
Van Heckeren, Dr. his differtation on morbific offification 76
Van Norden and Roupen's experiments 469
Van Mons, Cit. his preparation of the new falt difcovered by Chauffier 466
——— his diftillation of the oxygenated pomatum 470
Variolous contagion, Franks on 518

Vaume, Dr. on vaccine inoculation 450

Veitch on fracture of the skull 513

————————, addition to his case 572

———'s letter to the commissioners of sick and wounded seamen, on vaccine inoculation 309

Venereal complaints, Girtanner on the use of oxygen in 469

Vesiculae seminales, Crowther, Dr. on the induration of them 507

Vinegar, Simons, Dr. on the distillation of 411

Ulcer, Ballard on a particular species of 408

University of Cambridge, in America, sketch of it 569

Urethra, Aberdour on strictures of 387

Urinary, stones and gravel, Prof. Mascagni's observations on 467

Uwins on febrifuge medicines 53

Waiblinger, his case of dislocated patella 285

Wainwright on the belladonna 5

Ward on the treatment of hernia 37

Water, putrid, Lucas on making it potable 87

———, cold, Dr. Beer on its application in amaurosis (*with a plate*) 179

—————, Reeve on the affusion of 397

Waterhouse, Dr. his account of the vaccine inoculation at Cambridge, in New England 471

————— on the kine-pox 563

Waters, mineral, of Driburg, in Westphalia, Dr. Cappel on 87

—————, Dr. Saunders's treatise on announced 91, 350

Waters, Bath, Gibbes, Dr. on 457

Waters, Mineral, Brugnatelli's new arrangement of 377

Watherston on the citric acid in sea scurvy 155

Watt in reply to Sheldrake, on distorted limbs 498

Watt, his description of a new machine for curing distorted limbs 93

———, Sheldrake in answer to, on distorted limbs 192

Werner, Dr. his apology for the Brunonian system 275

West Indies, Fowle's, Dr. treatise on fevers in 354

Westminster Hospital, account of diseases in 14, 107

Westphalia, mineral waters at Driburg in, Dr. Cappel on 87

Wichmann, Dr. his theory of difficult dentition in children 171, 269

Willan, Dr. his observations on the diseases of London announced 4[illegible]1

Wind in the intestinal tube, Prof. Ackermann's dissertation on 556

Wolcott, Dr. his treatise on the general cause of deafness announced 187

Wood, rotten, Humboldt on the illumination of 85

Woodward on infantile diseases 42

Wollaston, Dr. on the danger of inoculating with purulent fluids 488

Wurzer, Prof. on the use of nitric acid in a case of lues venerea 138

Yeast in typhus, Cuftance on 17

—————, Grose on 953

—————, and cold affusion, Grose on the use of 401

### *References to the Plates.*

Page

Humboldt's Anthracometer - - - - 93

Watt's Machine for Curing Distorted Limbs - - ib.

Dr. Beer's Instrument for applying cold Water in Amaurosis 179

Sketch of a monstrous Foetus - - - - - 189

Waiblinger's Bandage for a Dislocated Patella - - 285

Prof. Arnemann's Acoustic Tubes - - - 378

Ford's Case of a Diseased Bladder with Enlargement of the Prostate Gland - - - - - 381

Cases of Dysphagia - - - - - - 477

Sheldrake's Plates of Distorted Feet - - 493, 494

Printed by William Thorne, Red-Lion-Court, Fleet-Street.

